# Shigella Diversity and Changing Landscape: Insights for the Twenty-First Century

**DOI:** 10.3389/fcimb.2016.00045

**Published:** 2016-04-19

**Authors:** Mark Anderson, Philippe J. Sansonetti, Benoit S. Marteyn

**Affiliations:** ^1^Institut Pasteur, Unité de Pathogénie Microbienne MoléculaireParis, France; ^2^Institut National de la Santé et de la Recherche Médicale, Unité 786Paris, France; ^3^Collège de FranceParis, France

**Keywords:** Shigella, diversity, virulence, microbiota, mucus

## Abstract

Shigella is a pathovar of *Escherichia coli* comprising four groups, *Shigella flexneri, Shigella sonnei, Shigella dysenteriae*, and *Shigella boydii*, each of them, with the exception of *S.*sonnei, comprising several serotypes. Shigella accounts for the majority of dysentery causing infections occurring world-wide each year. Recent advancements in the Shigella field have led to a better understanding of the molecular mechanisms underlying host epithelial cell invasion and immune cell function manipulation, mainly using *S. flexneri* as a model. Host-cell invasion is the final step of the infection process, as Shigella's virulence strategy relies also on its ability to survive hostile conditions during its journey through the gastro-intestinal tract, to compete with the host microbiota and to cross the intestinal mucus layer. Hence, the diversity of the virulence strategies among the different Shigella species has not yet been deeply investigated, which might be an important step to understand the epidemiological spreading of Shigella species worldwide and a key aspect for the validation of novel vaccine candidates. The recent development of high-throughput screening and sequencing methods will facilitate these complex comparison studies. In this review we discuss several of the major avenues that the Shigella research field has taken over the past few years and hopefully gain some insights into the questions that remain surrounding this important human pathogen.

## Introduction

Shigella are Gram-negative pathogenic enterobacteria and the etiological agent of bacillary dysentery or shigellosis. In humans, Shigella specifically invade and colonize the colonic mucosa leading to its disruption. Shigellosis is associated with fever and abdominal cramps; its diagnosis relies on the presence of erythrocytes, polymorphonuclear neutrophils (PMNs), and mucus in patient stools, which stand as diagnostic elements.

**Figure 1:**
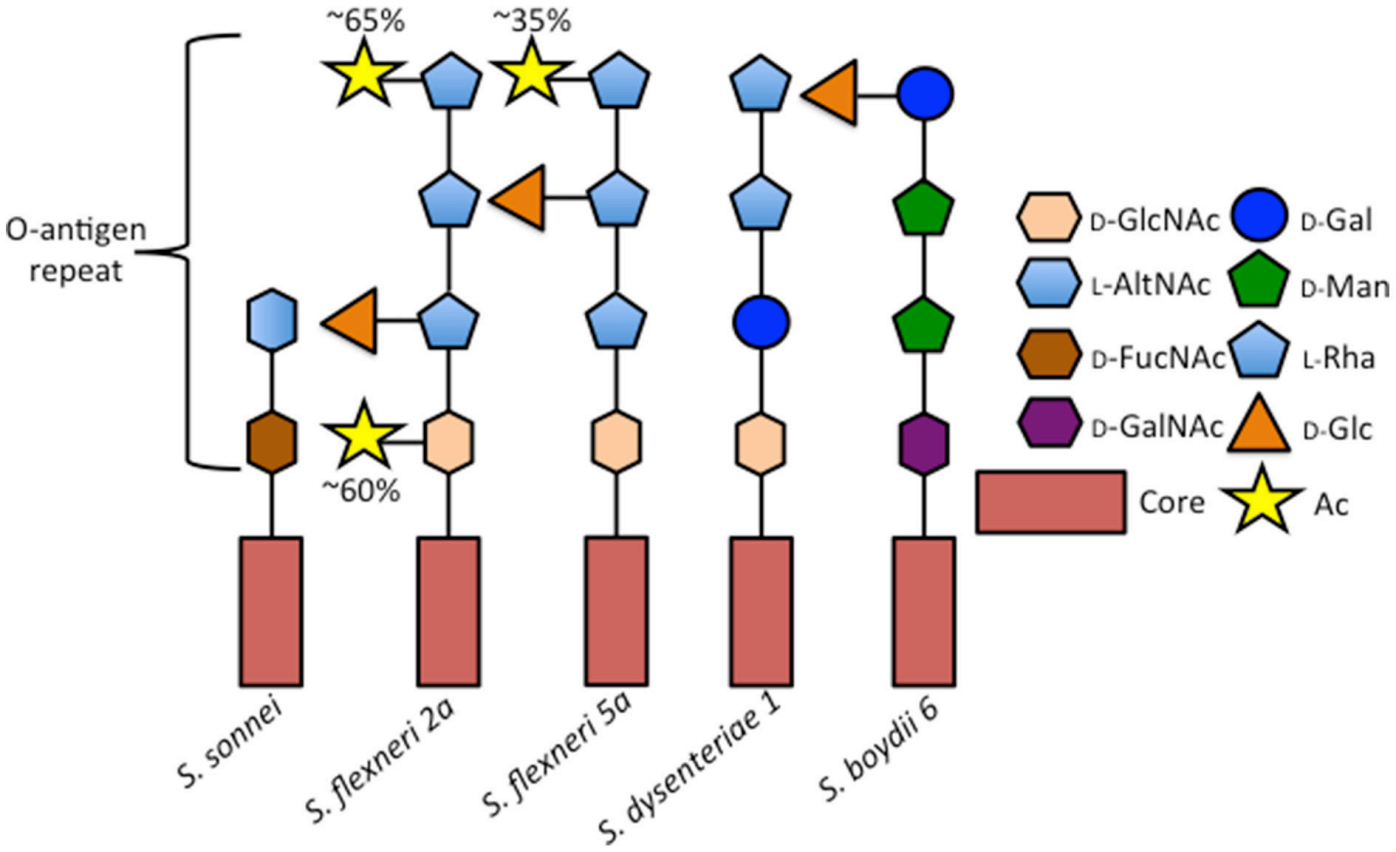
**Comparison of selected Shigella O-antigen side chains**. Schematic of repeated O-antigen side chains from serotypes *S. sonnei* (Gamian and Romanowska, 1982), *S. flexneri* 5a (Perepelov et al., 2010), *S. dysenteriae 1* (Dmitriev et al., 1976), and *S. boydii* 6 (Senchenkova et al., 2005). Figure legend abreviations are as follows 2-Acetamido-2-deoxy-D-glucose (D-GlcNAc), 2-Acetamido-2-deoxy-L-altruronic acid (L-AltNAc), 2-Acetamido-4-amino-2, 4-dideoxy-D-fucose (D-FucNAc), 2-Acetamido-2-deoxy-D-galacturonic acid (D-GalNAc), D-Galactopyranose (D-Gal), D-Mannose (D-Man), L-Rhamnose (L-Rha), D-Glucose (D-Glc), *O*-acetyl (Ac).

Shigella encompasses four subgroups (*S. flexneri, S. sonnei, S, dysenteriae*, and *S. boydii*), each composed of different serotypes, which are identified based on the structure of the lipopolysaccharide O-antigen repeats: *S. dysenteriae* encompasses 15 serotypes, *S. flexneri*, 14 serotypes, *S. boydii*, 20 serotypes, and *S. sonnei* a single serotype (Figure 1; reviewed in Marteyn et al., 2012). Chantemesse and Widal first described the bacillus causing non-amoebic dysentery in 1888, while Shiga first identified *S. dysenteriae* in 1898 (Shiga, 1898). Flexner first characterized *S. flexneri* in 1900 and *S. sonnei* was isolated and characterized by Sonne (1915). *S. boydii* was first described by Boyd in 1931.

Studies of host-pathogen interactions have mostly been restricted to the direct contact with and invasion of the host epithelial and immune cells. This is particularly true in the Shigella field. Its adhesion and invasion of host cells and its intracellular life have been characterized at a molecular level (reviewed in Tran Van Nhieu et al., 2000; Phalipon and Sansonetti, 2007). In this review, we envisage the host-pathogen interaction with a more global perspective, considering that prior to challenging epithelial or immune cells, Shigella must first interact and compete with the microbiome and subsequently face a thick mucus layer protecting the colonic epithelium. Shigella virulence mechanisms involved in these latter aspects have been under evaluated, although they represent key and essential features of a successful infection process.

Shigella virulence mechanisms have been mostly identified and characterized using *S. flexneri* as a model, while other groups show significantly high prevalence worldwide. In this review, we will emphasize the importance of performing comparative studies to better understand and characterize Shigella pathogenicity. The development of novel genetic tools and high-throughput screening and sequencing methods permits the analysis of Shigella virulence mechanism diversity.

## Shigella diversity: epidemiology, geographical distribution, and emerging antibiotic resistance

Shigella remains a leading cause of childhood morbidity and mortality. The recently conducted case-controlled Global Enteric Multicenter Study (GEMS) provided a solid update on the incidence of Shigella among severe forms of diarrhea, and convincingly demonstrated that in the sites considered (Sub-saharan Africa, and Asia), Shigella appeared amongst the top ranking pathogens identified (Kotloff et al., 2013).

Many observations have concluded that Shigella species are geographically stratified based on the level of economic development in a given country. *S. flexneri* is the primary infectious species in the developing world whereas *S. sonnei* rates increase with economic development. *S. boydii* is most commonly restricted to Bangladesh and South-East Asia and rarely occurs outside of these regions. *S. dysenteriae* type 1 (Sd1) occurs sporadically in outbreak settings with striking examples occurring in refugee camps during the civil war in Rwanda between November 1993 and February 1995 in which more than 180 thousand cases and significant mortality from Sd1 were recorded (Kernéis et al., 2009). The last major Sd1 outbreak occurred in 1999 during the civil war in Sierra Leone resulting in over 4000 cases (Guerin et al., 2004), and the cyclic occurrence of Sd1 in Bangladesh every 10 years is clearly discontinued for unknown reasons, illustrating major remaining uncertainties on changes of epidemiological patterns of these infections.

**Figure 2:**
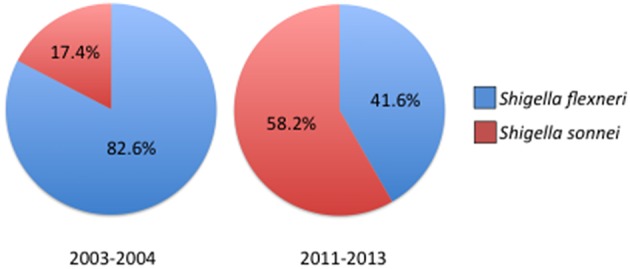
**Prevelance of Shigella species across China 2003–2013**. Comparison of reported Shigella species between 2003–2004 and 2011–2013 showing the increase in *S. sonnei* isolations. For years 2003–2004 *n* = 235 cases, for 2011–2013 *n* = 1049 cases. Prevelance data reported in Qiu et al. (2015).

Outside of outbreak settings, *S. flexneri* and *S. sonnei* account for the majority of Shigellosis cases. Recent epidemiological studies conducted around the world have discovered a rise in the proportion of *S. sonnei* isolates compared to *S. flexneri*. The expansion of *S. sonnei* can clearly be observed from clinical surveillance studies conducted in China which show the proportion of *S. sonnei* isolates increasing from 17.4% in 2003–2004 to 58.2% less than a decade later, closely following the rapid industrialization in China (Figure 2; Mao et al., 2013; Qiu et al., 2015). Noticeably, regions that had undergone significant industrialization reported decreases in *S. flexneri* and increasing cases of *S. sonnei* compared to under developed areas where flexneri levels remain high (Qiu et al., 2015). Rising cases of *S. sonnei* have also been detected in Bangladesh, which has historically been affected by all four species of Shigella. Between 2001 and 2011 the proportion of *S. sonnei* infections rose from 7 to 25% of reported cases in Bangladesh, which also corresponds with enhanced sanitation and clean water efforts throughout the country (Das et al., 2013; Hulland et al., 2013). The reasons for the counter-intuitive increase of *S. sonnei* in the face of better sanitation have not been determined, however several hypotheses have been put forward (Thompson et al., 2015). *S. sonnei* and *Plesiomonas shigelloides* share a common O-antigen that may lead to natural cross-protective immunity in populations that encounter high levels of *P. shigelloides* due to contaminated water supplies (Sack et al., 1994). Other avenues of interest include observations of increased survival and replication of *S. sonnei* in *Acanthamoeba* which may act as a reservoir and increased ability to acquire antibiotic resistances (Saeed et al., 2012). As more countries increase their level of development and sanitation it is likely that *S. sonnei* will become even more of a global public health concern which could have important impacts on vaccine development efforts.

A major concern surrounding Shigella is its capacity to rapidly acquire antibiotic resistances. Development of resistance to antibiotics is common in all Shigella species, particularly in *S. sonnei*, which can acquire resistance genes directly from *E. coli* through horizontal gene transfer. Several recent reports have suggested that *S. sonnei* is capable of sharing resistance plasmids through conjugation with commensal *E. coli* (Qu et al., 2014; Rashid and Rahman, 2015). Recently, *S. sonnei* resistance to the frontline antibiotic, ciprofloxacin, has been associated with travel to Asia and India and has been imported to the United States (Bowen et al., 2015; De Lappe et al., 2015; Kim et al., 2015). Preventing the global spread of resistant *S. sonnei* strains presents a significant public health challenge as intercontinental travel has become common.

Antibiotic resistance in *S. flexneri* is also well-documented with many studies finding high rates of resistance to at least one common antibiotic such as ampicillin, tetracycline, and chloramphenicol (Khaghani et al., 2014; Cui et al., 2015). These studies show that without access to proper testing facilities or multiple different antibiotics, patients affected by *S. flexneri* in developing countries have a high chance of not being successfully treated.

Two articles have shed some light on the expansion and spread of antibiotic resistance in Shigella. Sequencing and phylogenetic studies have pinpointed the emergence of *S. sonnei* from a single European clone that spread pandemically to multiple continents, diversifying and acquiring multiple antibiotic resistances along the way (Holt et al., 2013). The sequencing data also suggest that antibiotic resistant *S. sonnei* strains have been exported to new areas via infected travelers. In contrast, Connor et al. recently published results from a whole genome sequencing study of *S. flexneri* that found considerable independent acquisitions of antibiotic resistance yet little global spread of resistant flexneri strains, instead favoring a model of local resistance acquisition and persistence rather than pandemic spread (Connor et al., 2015). These complementary studies have yielded the clearest information to date on how Shigella has spread throughout the global community. Taken together a prediction can be made that efforts to combat the spread of antibiotic resistance should focus more heavily on *S. sonnei* surveillance while more localized efforts are needed to combat resistance in *S. flexneri*.

The changing dynamics of *Shigella* species, particularly trends like the disappearance of Sd1 and transition from *S. flexneri* to *S. sonnei* raise important unanswered questions. Thus, far, the molecular and cellular mechanisms of *Shigella* pathogenesis have largely considered the invasion step and likely do not account for the major differences among the four subgroups. Instead, research probing the early steps of establishment/colonization may provide new insights into explaining the epidemiological observations.

## First barriers to shigella infection: the microbiota and the mucus layer

Shigella specifically invades the human colon, but not the small intestine. The molecular basis of this specificity remains unknown. Prior to reaching the epithelial lining, Shigella have to survive in the GI environment, to outcompete the colonic luminal commensal bacteria and to disrupt a thick protective mucus layer. For a long time, the mucus layer was considered as the first barrier to Shigella infection: it appears to be the second, the microbiota standing as the first. The inflammatory response induced by Shigella invasion might result in a perturbation of the composition and the function of these barriers, although these aspects are not yet well-described.

### First barrier: the microbiota

**Figure 3:**
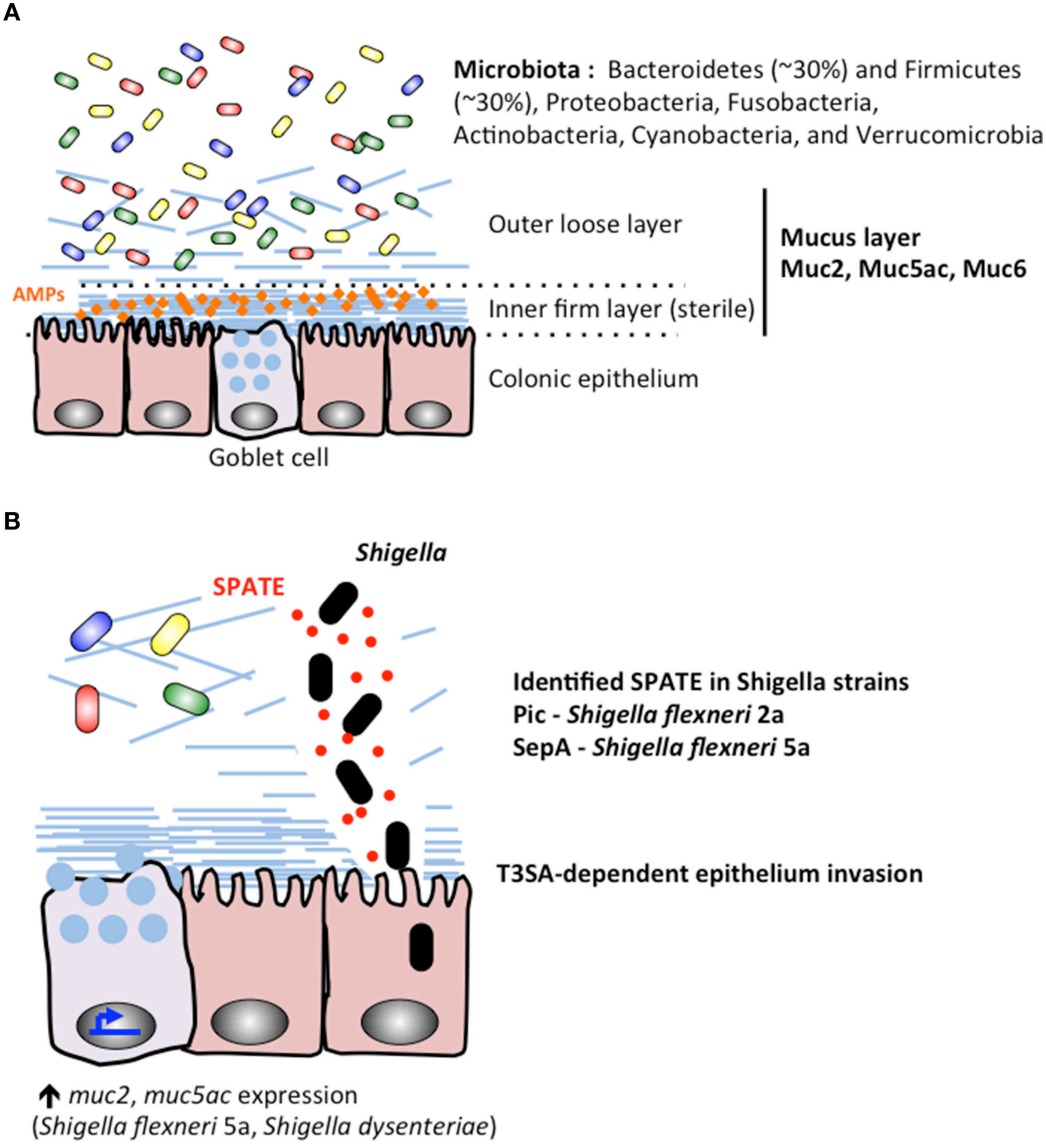
**During Shigella infection, the colonic microbiota and mucus layers stand as the first barriers protecting the epithelium lineage. (A)** The microbiota colonizes the colonic lumenal compartment, and the outer mucus layer. The inner mucus sterile remains sterile probaly due to its mechanical properties and accumulation of secreted antimicrobial peptides (AMPs). **(B)** Shigella secrete proteins belonging to the SPATE family, including *Pic* characterized in *S. flexneri 2a* (Henderson et al., 1999; Gutiérrez-Jiménez et al., 2008; Navarro-Garcia et al., 2010; Ruiz-Perez et al., 2011) and SepA identified in *S. flexneri 5a* (Benjelloun-Touimi et al., 1995). Shigella SPATE proteins cleave mucins to allow the bacteria to reach the epithelial layer. Shigella Type III secretion apparatus (T3SA) is required for epithelium invasion.

The gut microbiome of healthy individuals comprises the most complex interface between microbes and human tissues in the body. Over 1000 different species of bacteria reside at varying amounts and locations throughout the GI tract and 70% of the host immune system is dedicated to maintaining its integrity (Vighi et al., 2008; Rajilić-Stojanović and de Vos, 2014). The colonic microbiota can vary significantly between individuals but is dominated at the phyla level by Bacteroidetes (~30%) and Firmicutes (~30%), with lower levels of Proteobacteria, Fusobacteria, Actinobacteria, Cyanobacteria, and Verrucomicrobia (Bäckhed et al., 2005; Andersson et al., 2008; Arumugam et al., 2011; Sankar et al., 2015; Figure 3). The microbiota is further stratified based on proximity to the mucosal lining of the colon with genera such as Coriobacteriaceae, Lachnospiraceae, and Ruminococcaceae showing greater abundance in the mucus layer whereas Bacteroidaceae is more prevalent in the lumen of the intestine (Lavelle et al., 2015). Significant amounts of research have worked to characterize the role of the gut microbiome in both health and disease states and a picture of this complex community is beginning to take shape.

The study of how pathogens interact with and supplant host commensal bacteria is fast becoming an area of intense research. These interactions generally occur at the earliest stages of disease and set up the environment for a pathogen to gain entry into host tissues. Currently, very few studies have investigated the role of the microbiota in Shigella infections. The infectious dose of Shigella is thought to be quite low, (~10) organisms, so it is likely that Shigella has evolved some mechanism(s) to survive while being vastly outnumbered.

Early works assessed the role of individual commensal organisms in preventing the colonization of Shigella. Germ free mice and Guinea pigs monocolonized with *E. coli* were shown to block the establishment of *S. flexneri* whereas monocolonization with Bacteroides, Bifidobacterium, or Streptococcus had no effect on Shigella levels (Formal et al., 1961; Maier and Hentges, 1972). Other studies have shown treatment with streptomycin produces an environment that supports Shigella colonization in mice (Pongpech et al., 1989; Martino et al., 2005). Together these studies suggest a complex interplay between Shigella and commensal bacteria that are not easily reconciled with the low infectious dose observed in humans. It is possible that the overwhelming inflammatory environment produced during human disease masks more subtle and complex early interactions between Shigella and commensal bacteria. Revisiting these issues with newer techniques will be important in deciphering the role of host microbiota in Shigellosis.

The production and resistance to secreted small molecule inhibitors such as colicins mediate one common form of competition between enterobacteraceae. Colicins are small, secreted proteins that can have various cytotoxic properties against phylogenetically related bacteria and are are almost always carried on plasmids that encode both the colicin and its cognate immunity factor, reviewed in Cascales et al. (2007). Strains that are capable of producing a colicin are better able to compete against colicin sensitive strains over ecological niches. In the case of *S. sonnei*, it has been reported that up to 93% of tested strains in Kolkata, India, harbor at least one colicin-encoding plasmid which were shown to target closely related susceptible *E. coli* strains, which as previously mentioned, were important for restricting Shigella colonization (Calcuttawala et al., 2015). Meanwhile, the chromosomally encoded O-antigen chain length regulator WzzB was recently reported to contribute to colicin E2 resistance in *S. flexneri* (Tran et al., 2014). Regulation of the O-antigen chain length has an important impact during the intracellular phase of *Shigella* infection, but Tran et al. suggest the exact number of repeat units has evolved to help mediate colicin resistance as well (Morona et al., 2003; Tran et al., 2014). The production and resistance to colicins by Shigella may have an important role in its initial competition with the host microbiota.

A growing number of reports suggest that members of the lactobacilli genus may contribute to colonization resistance against Shigella strains by inducing an anti-inflammatory state or by blocking adherence to epithelial cells (Tien et al., 2006; Moorthy et al., 2010; Zhang et al., 2012). Specifically, proteins found in the S-layers of gram positive Lactobacillus species have been found to inhibit adhesion of several different pathogens including *Salmonella enteritidis, EHEC O157:H7*, and *S. sonnei* to tissue culture cell lines although the exact mechanism(s) remains to be discovered (Golowczyc et al., 2007; Johnson-Henry et al., 2007; Zhang et al., 2012). Further assessments of the interaction of commensal bacteria with Shigella will be important to gain a more complete picture of how beneficial bacteria aid in host defenses against pathogens. Similarly, studies probing how Shigella overcomes the resident microbiota during successful infection would be of great interest.

### Second barrier: the mucus layer

A complex layer of mucus naturally protects human epithelium in the gastrointestinal tracts (stomach, small intestine, colon; Ambort et al., 2012). The role of the mucus layer is to prevent direct interaction between commensal bacteria and epithelial cells (Johansson et al., 2011), avoiding further stimulation of the immune system (Pelaseyed et al., 2014; Figure 3 upper panel). In addition to the barrier effect created by the mucus layer, antimicrobial peptides (AMPs) secreted by epithelial and phagocytic cells accumulate in the mucus layer and act as a further inhibitory blockade against microbial species (Meyer-Hoffert et al., 2008). Mucus is mainly composed of mucins (20 identified mucins in humans) reviewed in McGuckin et al. (2011), which are high-molecular weight (1–10 million Da) glycoproteins linked with each other through intermolecular disulfide-bonds between cysteine-rich D domains located in mucins amino and carboxyl termini. Mucins are either oligomeric, soluble and secreted (MUC2, MUC5ac, MUC5b, MUC6, and Muc7) or are membrane tethered, which might be associated with different functions. Mucins are naturally produced and released on epithelial surfaces: mainly by goblet cells but also (in a lesser extent) by epithelial cells. It has been recently shown that the surface colonic goblet cells secrete continuously, while the secretion of goblet cells located in the small intestine or colonic crypts are inducible (Birchenough et al., 2015). The thickness of the mucus layer varies along the GI tract in humans and various animal models (Varum et al., 2012). While the ileal mucus layer is thin (< 300 μm), the thickness of the colon mucus layer can reach 1 mm in humans. The colonic mucus layer is organized in two layers: the inner layer (50 μm) is firmly attached to the epithelium, the outer loose layer (>100 μm) is unattached (Figure 3). Several studies have described the ability of commensal bacteria to colonize and adhere to the abundant O-glycans present in the outer mucus layer, while the inner mucus layer remains sterile, due to its mechanical properties or capacity to retain secreted antimicrobial compounds, avoiding further stimulation of epithelial cells by commensal bacteria (Johansson et al., 2011). The sterility of the inner mucus layer might be altered by impairment of AMP production leading to colitis or other inflammatory bowel conditions (Kocsis et al., 2008; Johansson et al., 2014). The colonic mucus layer backbone is mainly composed of MUC2 (Karlsson et al., 1996), associated with MUC5AC and MUC6 (reviewed in McGuckin et al., 2011; Figure 3). Differential MUC2 glycosylation profiles are described in the ileum and in the colon, being enriched in either sialilated or sulfated oligosaccharide species respectively (Karlsson et al., 1996; Robbe et al., 2003). It has been shown that *S. dysenteriae* 1 adheres to human colonic mucins, not to small intestine mucins (Sudha et al., 2001), although no specific adhesion molecule has been identified.

Shigella efficiently invade the colon, which is protected by the thickest layer of mucus in the GI tract. In addition, it has been shown that *S. dysenteriae* infection induces the expression of MUC2 and MUC5AC *in vitro* in an IL-1β dependent-manner (Prakash et al., 2011; Raja et al., 2012), but also *in vivo* in a rat ileal loop model (Gopal et al., 2014). Similarly, *S. flexneri* induces MUC2, MUC5AC, MUC4, and MUC15 expression *in vitro* (HT29-MTX cells; Sperandio et al., 2013). Together with the fact that Shigella are devoid of flagella (which promote the mobility of various pathogens, such as *Helicobacter pylori* (Ottemann and Lowenthal, 2002), it suggests that Shigella express and releases mucolytic molecules allowing its progression through the mucus layer.

It has long been known that the degradation of the mucus outer layer and the release of mucin glycans, by glycoside hydrolase-expressing bacteria composing the microbiota, were used as a source of nutrients and energy (Deplancke et al., 2002; Sonnenburg et al., 2005). So far, no report describes similar adaptation of Shigella in the colonic lumen to promote its proliferation.

Mucus integrity can be altered by the several classes of enzymes secreted by enterobacteria (Figure 3 lower panel). It includes mucinases, glycosidases, sulfatases, sialidases (or neuraminidases), and sialilate O-acetylesterases (SIAE; Corfield et al., 1992; reviewed in McGuckin et al., 2011; Corfield, 2015). It was shown by Haider and colleagues that all four Shigella species were able to produce mucinases and neuraminidases (Haider et al., 1993), although since this initial observation no comparative study was performed. *Pic*, a *S. flexneri* 2A (4257T) autosecreted protein belonging to the SPATE (Serine Protease Autotransporters of Enterobacteriaceae) was characterized as a mucinase (Henderson et al., 1999; Gutiérrez-Jiménez et al., 2008). It was shown that the deletion of *pic* in *S. flexneri* 2a clinical isolates reduced its pathogenicity (Navarro-Garcia et al., 2010; Zhang et al., 2013). *Pic* homologous protein is expressed by *S. sonnei*, not in *S. flexneri 5a*, which instead secretes another protein belonging to the SPATE family (SepA), whose function remains unknown (Benjelloun-Touimi et al., 1995, 1998). *S. dysenteriae, S. flexneri 2a, S. sonnei* and *S. boydii* express homologous proteins to *Enterotoxigenic E. coli* (ETEC) EatA, belonging to the SPATE family, which has been shown to degrade MUC2 (Kumar et al., 2013). Until now, the role of EatA in Shigella virulence mechanisms has not yet been characterized.

After traversing the mucus layer, Shigella contact and inject epithelial cells with effector molecules that down-regulate production of AMPs, supporting an environment that is more conducive to further penetration into the tissue and providing evidence that AMPs are an important host defense factor against Shigella (Sperandio et al., 2008; Gudmundsson et al., 2010).

Altogether, these results suggest that Shigella potentially express and secrete mucinases, although the specificity of these enzymes will have to be characterized and their cytotoxicity further studied. It would also be of great interest to study the consequences of disrupting of the mucus layer on local AMP concentrations and inhibition of invading Shigella bacteria. Disruption of the mucus layer and dispersion of AMPs could play a significant role in Shigella survival in addition to aiding colonization.

## Global tools to decipher shigella virulence mechanism diversity

The advent of affordable, high-throughput sequencing technologies is allowing the field of microbiology to design powerful experiments that have previously been impossible to perform due to time, money, or computing restraints. With this technology however has come the very real problem of how to adequately mine the vast quantities of data generated for important information. In the Shigella field there has been an explosion in the number of sequenced strains and phylogenetic trees establishing a detailed history of the evolution of this pathogen. The most sophisticated sequencing analysis of *S. sonnei* to date comprised of 132 strains spread across four continents has yielded very interesting information on its origins and recent global spread (Holt et al., 2012). In this paper the authors estimate the most recent common ancestor to *S. sonnei* arose in Europe less than 500 years ago between 1554 and 1763 A.D (Holt et al., 2012). Additionally, it was found that the global spread of *S. sonnei* has only begun quite recently, since the mid twentieth century (Holt et al., 2012). A subsequent massive sequencing study of 263 *S. sonnei* strains, isolated over 15 years in Vietnam, has been used to track its evolution and to date its first arrival in the country to the 1980's (Holt et al., 2013). The result is a fascinating “high resolution” view of local adaptations and evolutionary changes that have occurred over the past 15 years from what started as a single imported clone (Holt et al., 2013). Sequencing studies like these are providing invaluable information about how pathogens evolve, expand, and become fixed in a population. However, the massive amount of data generated from sequencing so many isolates can present a daunting challenge to mine for insights into the functional outcomes of microbial evolution. Holt et al.'s (2012) paper alone reported detecting 10,111 SNP mutations in the sequenced strains making it difficult to investigate individual mutations for their impact on the fitness of Shigella. Ideally, it would be intriguing to assess Shigella's pathogenic traits over time based upon its evolution.

Alternatively, high-throughput sequencing has found another important use in new generations of mutant library design and screening applications. In *Vibrio cholerae*, pooled transposon mutant libraries have been screened in rich media and under *in vivo* conditions, coupled with next generation transposon sequencing (TN-seq) resulting in a global picture of genes essential for survival in a rabbit animal model (Kamp et al., 2013). This global approach allowed over 100,000 individual mutants to be screened simultaneously in a single model system, creating a nearly complete picture of the genes required for *V. cholerae* to survive in a host (Kamp et al., 2013). This technique has also successfully been demonstrated in the gram positive pathogen *Staphylococcus aureus*, where over 71,000 transposon mutants were screened *in vivo* (Valentino et al., 2014). Adapting this protocol to Shigella, coupled with the newly developed Guinea pig colonic infection model could give significant insights into the repertoire of genes needed to survive and replicate in the colon during infection (Arena et al., 2015). Furthermore, performing such experiments in representative *S. sonnei* and *S. flexneri* strains could allow for an interesting comparison study, highlighting similarities, and differences in growth and survival strategies between Shigella species which up until now have been impossible to perform.

## Conclusions

For several decades, *S. flexneri* strains (2a and 5a) were used as model organisms to characterize Shigella virulence mechanisms. However, each Shigella groups and subgroups exploit various virulence mechanisms and as a consequence deserved a specific analysis. The development of high-throughput technologies represents a key opportunity to address this fastidious but essential question without bias. It will allow comparative studies and a better comprehension of various Shigella group specific virulence features. This aspect is well-illustrated by the diversity of SPATE proteins expressed and secreted by different groups (see Section First Barriers to Shigella Infection: The Microbiota and the Mucus Layer), coming studies will probably highlight additional specificities.

The diversity of Shigella groups identified worldwide has changed over the last decades. Efforts to improve water sanitation have lead to a decrease in *S. flexneri* infections however *S. sonnei* rapidly moves in replacing *S. flexneri*. For this reason improving water supplies alone is not sufficient to eliminate the threat of Shigella. Vaccines targeting *S. sonnei* and remaining *S. flexneri* serotypes (i.e., 2a, 3a, and 6) +/− *S. dysenteriae* 1 must be part of a long-term strategy that takes in account the serotype-specificity of immune protection against Shigella, to reduce the global dysentery burden. However, the development of live, rationally-attenuated, oral vaccine candidates against Shigellosis has been handicapped by two major issues. (i) The difficulty to assemble a multivalent set of these epidemiologically-relevant serotypes, in the absence of a definitely-established cross-protective antigen (Levine et al., 2007). (ii) A clear discrepancy in the colonization and potential protective capacity of the so-far tested candidates when tested in western volunteers and in individuals living in endemic areas (Coster et al., 1999; Rahman et al., 2011).

Clearly, more basic research aimed at better understanding the basic mechanisms of Shigella colonization is warranted to master this key step and improve the design of future live-attenuated orally-administered vaccines.

## Author contributions

All authors listed, have made substantial, direct and intellectual contribution to the work, and approved it for publication.

### Conflict of interest statement

The authors declare that the research was conducted in the absence of any commercial or financial relationships that could be construed as a potential conflict of interest.
